# Genome-wide association mapping in a diverse spring barley collection reveals the presence of QTL hotspots and candidate genes for root and shoot architecture traits at seedling stage

**DOI:** 10.1186/s12870-019-1828-5

**Published:** 2019-05-23

**Authors:** Adel H. Abdel-Ghani, Rajiv Sharma, Celestine Wabila, Sidram Dhanagond, Saed J. Owais, Mahmud A. Duwayri, Saddam A. Al-Dalain, Christian Klukas, Dijun Chen, Thomas Lübberstedt, Nicolaus von Wirén, Andreas Graner, Benjamin Kilian, Kerstin Neumann

**Affiliations:** 1 Department of Plant Production, Faculty of Agriculture, Mutah University, Mutah, Karak, 61710 Jordan; 2 Leibniz Institute of Plant Genetics and Crop Plant Research (IPK), Corrensstrasse 3, 06466 Seeland, Germany; 3 Division of Plant Science, University of Dundee at JHI, Invergowrie, Dundee, DD2 5DA UK; 4 Department of Horticulture and Agronomy, Faculty of Agriculture, University of Jordan, Amman, Jordan; 5 Al-Shoubak University College, Al-Balqa’ Applied University, Al-, Salt, 19117 Jordan; 6 Digitalization in Research & Development (ROM), BASF SE, 67056 Ludwigshafen, Germany; 7 Department for Plant Cell and Molecular Biology, Institute for Biology, Humboldt University Berlin, 10115 Berlin, Germany; 8 Department of Agronomy, Agronomy Hall, Iowa State University, Ames, IA 50011 USA; 9 Martin-Luther-University Halle-Wittenberg, Betty-Heimann-Str. 3, 06120 Halle/Saale, Germany; 10 Global Crop Diversity Trust, Platz der Vereinten Nationen 7, 53113 Bonn, Germany

**Keywords:** Barley, Candidate genes, Genome-wide association study, Osmotic stress, Root architecture

## Abstract

**Background:**

Adaptation to drought-prone environments requires robust root architecture. Genotypes with a more vigorous root system have the potential to better adapt to soils with limited moisture content. However, root architecture is complex at both, phenotypic and genetic level. Customized mapping panels in combination with efficient screenings methods can resolve the underlying genetic factors of root traits.

**Results:**

A mapping panel of 233 spring barley genotypes was evaluated for root and shoot architecture traits under non-stress and osmotic stress. A genome-wide association study elucidated 65 involved genomic regions. Among them were 34 root-specific loci, eleven hotspots with associations to up to eight traits and twelve stress-specific loci. A list of candidate genes was established based on educated guess. Selected genes were tested for associated polymorphisms. By this, 14 genes were identified as promising candidates, ten remained suggestive and 15 were rejected. The data support the important role of flowering time genes, including *HvPpd-H1*, *HvCry2*, *HvCO4* and *HvPRR73*. Moreover, seven root-related genes, *HERK2, HvARF04, HvEXPB1, PIN5, PIN7, PME5* and *WOX5* are confirmed as promising candidates. For the QTL with the highest allelic effect for root thickness and plant biomass a homologue of the Arabidopsis *Trx-m3* was revealed as the most promising candidate.

**Conclusions:**

This study provides a catalogue of hotspots for seedling growth, root and stress-specific genomic regions along with candidate genes for future potential incorporation in breeding attempts for enhanced yield potential, particularly in drought-prone environments. Root architecture is under polygenic control. The co-localization of well-known major genes for barley development and flowering time with QTL hotspots highlights their importance for seedling growth. Association analysis revealed the involvement of *HvPpd-H1* in the development of the root system. The co-localization of root QTL with *HERK2, HvARF04, HvEXPB1, PIN5, PIN7, PME5 and WOX5* represents a starting point to explore the roles of these genes in barley*.* Accordingly, the genes *HvHOX2, HsfA2b, HvHAK2,* and *Dhn9,* known to be involved in abiotic stress response, were located within stress-specific QTL regions and await future validation.

**Electronic supplementary material:**

The online version of this article (10.1186/s12870-019-1828-5) contains supplementary material, which is available to authorized users.

## Background

Drought stress is the principal constraint of barley production in West Asia and North Africa (WANA) [1, 2] with only one third of the yield compared with Europe [3], mainly because of low (< 300 mm) and unpredictable season-to-season inter-variability in rainfall. Improving crop performance and grain yield and stability under drought is a major goal of plant breeding programs targeting these regions. Crops can be exposed to drought during their entire life cycle from vegetative to reproductive stages [4–6]. Water shortage can cause severe problems to seedlings, restricting the emergence of seedlings, seedling growth and development and thus affecting grain yield [7, 8]. Vigorous root systems are often considered as a primary target to breed for drought tolerance [9–13]. The importance of root traits as indirect selection criteria to increase yield was revealed by recent studies showing a significant positive relationship between root traits at seedling stage and grain yield under drought conditions in barley [14–17], wheat [18, 19] and in maize [5].

To assess root architecture traits in the field, vertical root pulling force (RPF) has been used, which is laborious, technically demanding and of insufficient precision [20–22]. As alternatives, high-throughput laboratory screens for root architecture evaluation, such as hydroponic or gel chamber-based systems [23–26] have become available in conjunction with imaging-based methods allowing for simultaneous analysis of multiple traits [27–30]. Robust high-throughput laboratory screens can be used to screen large numbers of genotypes within a short period and limited space [31–34].

In addition, root traits are easier to be assessed under control versus stressful growth conditions [2, 11, 35]. To date, most genetic studies on drought stress were based on visual phenotyping of above-ground plant parts in field studies [36–40]. Assessing root traits in hydroponically-grown seedlings is a valid approach for genetic studies, especially when large numbers of individuals are required for reliable trait quantification [41, 42]. Genome-wide association studies (GWAS) represent a powerful method for studying complex traits in cereals [43–46]. A meta-QTL analysis in rice identified 119 QTL for 29 root architecture traits from 24 studies distributed over the whole rice genome, indicating a complex inheritance of root traits [47]. In line with [47], QTL studies indicate a complex nature of root architecture being under polygenic control in barley [e.g. [21, 33, 48–51] ], wheat [19, 52–54] and maize [55–57]. Root architecture can also be influenced by abiotic stress but only few studies addressed this topic [21, 49, 58, 59]. Further, we found no report about any root QTL in barley specific for drought conditions.

There is only limited knowledge in barley of candidate genes known to influence root growth. Auxin as a plant hormone plays a role in growth throughout various developmental stages and is involved in root initiation as well as cell division and extension, and regulates gene expression for instance, via auxin response factors (ARFs) [60]. Posttranslational modification of ARF7 was shown to regulate root branching in Arabidopsis [61]. Recently, twenty ARFs were identified in the barley genome [62]. A well-known example for a root architecture gene in monocots is *DRO1* in rice that regulates root angle and was discovered by positional cloning of a QTL for deep rooting [63]. In barley, the gene *HvEXPB1* has been recently described to cause root hair initiation encoding the cell wall-loosening protein EXPANSIN [64]. The barley gene *HvWAK1* is encoding a cell wall-associated receptor-like kinase, which are essential for growth and development. It was shown that its expression is root-specific and that *HvWAK1* mutants differed in root growth under non-stress and salt stress conditions compared to the wildtype [65].

Further genes with an influence on both root and shoot growth are the *Rht* dwarfing genes in wheat [66], the semi-dwarfing genes *sdw1* and *ari-e.GP* in barley [67] and also *VRN1* in both crops [68].

The high heritabilities of seedling root and shoot characteristics obtained in hydroponic experiments [69] in a diverse spring barley panel paved the way to employ GWAS to study their genetic architecture. Benefitting from recent achievements in barley genomics [70] GWAS can be efficiently combined with genomic information to discover candidate genes associated with root developmental traits.

The objectives of this study were (i) to investigate the phenotypic variation of selected root and seedling traits in a diverse panel of spring barley genotypes, (ii) to unravel their genetic architecture by performing GWAS based on a large array of SNP markers and (iii) to identify candidate genes underlying hotspots of Quantitative Trait Loci (QTL) under two contrasting water availability treatments.

## Methods

### Mapping panel

A total of 233 spring barley genotypes was used for phenotypic analysis (Additional file 1: Table S1). Most barley lines (223) originate from a panel harboring broad genetic and phenotypic diversity that was successfully employed in genome-wide association scans for a variety of traits, for instance [46, 59, 71–73]. This panel was selected from IPK’s Genebank and was single seed descended twice. Additionally, eight modern two-rowed cultivars and two parents of a double-haploid (DH) population were included in this study. The total collection of 233 lines consists of 135 (58%) two-rowed and 98 (42%) six-rowed genotypes of world-wide origin [69].

### Experimental set-up with and without application of osmotic stress

Root architecture traits were measured in two independent experiments under variable water availability. The experimental procedure was described previously [69]. In short, four 3-day old seedlings for each genotype were placed on filter paper (size 30 × 20 cm) and wrapped into rolls. Rolls were maintained in Hoagland nutrient solution in the absence or presence of 15% w/v polyethylene glycol (PEG) 6000 in a growth chamber with 16/8 h of light at 20/18 °C at a light intensity of 200 μmol photons m-2 s-1 and 70% relative humidity. Within each treatment, two paper rolls per genotype were evaluated. Root and seedling traits were recorded 17 days after germination individually for three seedlings of each roll - except for root dry weight that was measured based on all roots from all three seedlings per roll due to their very low weights (Table 1). Two consecutive experiments were carried out. Each separate experiment was laid out in a randomized complete block design (RCBD) with split-plot arrangement of the two replications per treatment as main plot and genotype as sub-plot factor. Barley genotypes were randomized within the main plots.

**Table 1:** Trait list, abbreviations and description

Trait	Abbreviation^++^		Description	
	Non-Stress	Stress	Unit	
Root dry weight^+^	Rdwc	Rdws	mg	Roots were dried at 70 °C for 48 h and their weights were recorded using a weighing balance (Sartorius AC 1215, Germany).
Shoot dry weight^+^	Sdwc	Sdws	mg	Shoots were dried at 70 °C for 48 h and their weights were recorded using a weighing balance (Sartorius AC 1215, Germany).
Total plant biomass^+^	Byc	Bys	mg	Dry weights of shoots and roots were summed to get biomass/ biological yield of the seedlings.
Root to shoot ratio^+^	Rsc	Rss	ratio	It is measured as ratio of Root to Shoot dry weights.
Maximum root length^+^	Rlc	Rls	cm	Using scaled ruler maximum root lengths/ root length was measured.
Shoot length^+^	Slc	Sls	cm	Scaled ruler was used to record the shoot length of the seedlings.
Number of main roots	Nmrc	Nmrs	No.	Based on images of the scanned roots number of roots were recorded.
Average root thickness	Rthc	Rths	mm	Based on images of the scanned roots average root thickness were recorded.
Total root length	Trlc	Trls	mm	All roots were considered in recording the total root length based on images of the scanned roots.
Total root volume	Trvc	Trvs	mm^3^	All roots were considered in recording the total root volume based on images of the scanned roots.
Drought susceptibility index	DSI			It is calculated using the formula of Fisher and Maurer (1978) using Biological yield of control and stress see more details in Adel-Ghani et al. (2015).

^+^These traits were scored previously by Abdel-Ghani et al. (2015). ^++^C and S denotes the control and stress treatments

### Phenotyping of seedling trait architecture

Previously, we investigated seven traits from this seedling assay (maximum root length, root dry weight, shoot dry weight, total seedling biomass, root to shoot ratio, shoot length, and drought susceptibly index based on seedling biomass) [69].

For this study, four additional image-based traits derived from scanned seminal roots (Additional file 2: Figure S1) were analyzed: total root length, number of the main roots addressing seminal root number, total root volume and average root thickness (Table 1). All seminal roots of each seedling plant per filter paper roll (three per roll) were separated and placed into a flat bowl filled with water and scanned (Epson Expression 10,000 XL). The images had a resolution of 2044 × 2219 pixels. For image analysis, we used the automated root analysis pipeline implemented in the IAP software [74]. The analysis of the images followed four main steps: (i) pre-processing –images were prepared for segmentation, (2) segmentation – images were divided into different parts which have different meanings (for example, foreground – the root part; background – imaging chamber) with K-means based auto-tuning, (3) removal of small noise objects and root skeletonization, and (4) post-processing – to summarize calculated results for each root. More details are provided in [74].

### Statistical analysis of phenotypic traits

Analyses of variance (ANOVA) were calculated using the following model: *y*_*jki*_ *= μ + G*_*j*_ *+ E*_*i*_ *+ B*_*k(i)*_ *+ GE*_*ji*_ + ε_jki_*,* where *y*_*jki*_ represents the individual observation of the *jki*^*th*^ experimental unit, *μ* is the grand mean, *E*_*i*_ is the effect of *i*^*th*^ independent experiment, *B*_*k(l)*_ is the effect of *k*^*th*^ block nested in *i*^*th*^ experiment, *G*_*k*_ is the effect of *k*^*th*^ genotype, *GE*_*ji*_ is the interaction effect of the *i*^*th*^ experiment with *k*^*th*^ genotype, and ε_jki_ is the residual. The experiment was fitted as a fixed effect, whereas blocks and genotypes were fitted as random effects.

The phenotypic variance (${\sigma}_p^2\Big)$ was estimated according to the following formula: ${\sigma}_p^2={\sigma}_g^2+\frac{\sigma_{g\times ex}^2}{n}+\frac{\sigma_e^2}{n\times r}$,

where, ${\sigma}_g^2$ is the genotypic variance component, ${\sigma}_{g\times ex}^2$ is the genotype × experiment variance, ${\sigma}_e^2$ is the residual variance component, r is the number of replicates and n is the number of experiments. An estimate of the broad-sense heritability (*h*^*2*^) on plot basis was calculated as the ratio between the genetic (${\sigma}_g^2$) and the phenotypic (${\sigma}_p^2$) variance [75]. Pearson correlations between traits were calculated in R 3.5 [76].

### Genotypic data and genome-wide association scans

The whole barley panel in this study was genotyped using the 9 K iSelect array (Illumina, CA, United States) for barley containing 7864 SNPs [77]. After genotype calling, quality control filtering was applied, and markers with poor quality (> 10% of missing data) and minor allele frequency (MAF) less than 5% were excluded from further analysis, leaving 6019 high-quality SNPs. Among them, all mapped SNPs (4966; 82.5%) were considered for GWAS using the genetic map provided by [77]. Positions of mapped SNPs were further updated according to the Popseq map [78] if information was available.

The GWAS analysis was performed in the R package Genome Association and Prediction Integrated Tools (GAPIT version 2) [79]. Population structure using PCA and kinship was calculated in GAPIT and included in the GWAS model to control false positives. In total, three PCs were incorporated, explaining combined 26% of the total variation (individually: 16, 6 and 4%). The SUPER (settlement of MLM under progressively exclusive relationship) method [80] known to increase the statistical power was used to calculate genome-wide associations. To account for false positives due to multiple testing, the false discovery rate (FDR) method (FDR-adjusted, *P* < 0.05) [81] implemented in GAPIT was applied to the GWAS results. A schematic map indicating QTL positions was drawn using MapChart 2.2 Windows [82].

### Candidate gene evaluation

Candidate genes (CGs) were retrieved from the literature and by screening important QTL regions for annotated high confidence genes employing [83]. Further, the GBS data set of [84], which included the accessions employed in the present study, was screened for SNPs within these CGs and used for CG-association approach after filtering for MAF (> 0.05) and missing data (< 10%). Moreover, genomic fragments of twelve CGs were re-sequenced in the association panel. Genomic DNA was isolated from single leaves of each genotype with the Qiagen DNeasy Plant Mini Kit (Qiagen, Hilden, Germany), according to the manufacturer’s instructions. The Primer3 online software [85] was used to design PCR primers. Primers amplified one to two fragments for each CG (Additional file 1: Table S2). PCR products were purified by NucleoFast 96 PCR plates (Macherey-Nagel, Germany) and were sequenced directly on both strands on Applied Biosystems (Weiterstadt, Germany) ABI Prism 3730xL sequencer using BigDye terminators. DNA sequences were processed with AB DNA Sequencing Analysis Software 5.2 and later manually edited by Bio-Edit version 7.0.9.0 [86].

Sequence alignments were generated with ClustalW, and the allelic haplotypes were defined by DNASP 6.12 [87]. All singletons have been confirmed afterwards by additional three independent amplifications and sequencing.

## Results

### Phenotypic evaluation of seedling trait architecture under contrasting growth conditions

**Fig. 1:**
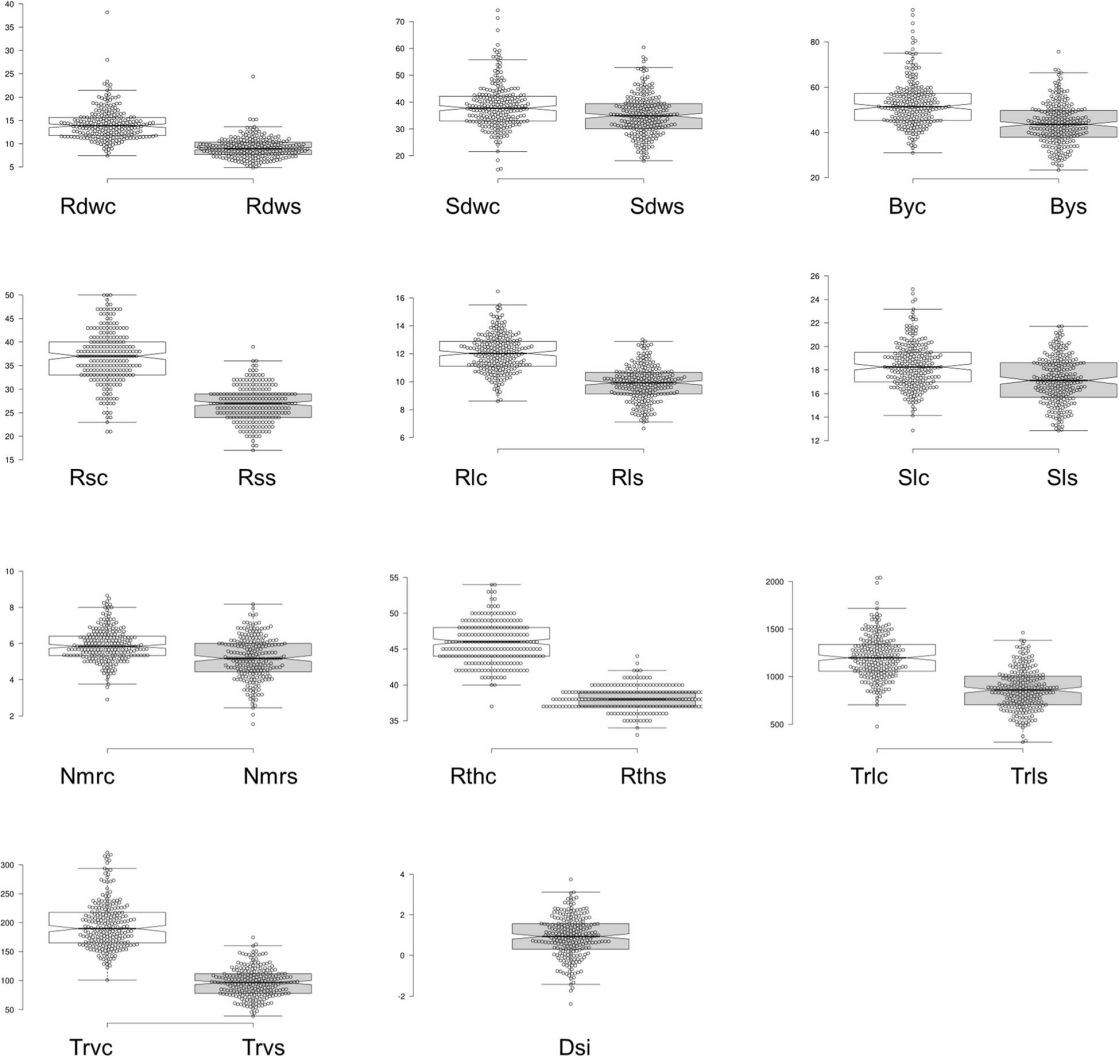
Box plots for root and seedling traits. Centre lines show the medians; box limits indicate the 25th and 75th percentiles as determined by R software; whiskers extend 1.5 times the interquartile range from the 25th and 75th percentiles, outliers are represented by dots; data points are plotted as open circles. Trait values for Rsc, Rss, Rthc and Rths are transformed by multiplying by 100 for visualizations

In total, ten traits were recorded under two different moisture treatments. Additionally, DSI was calculated and used as a derived trait for GWAS. A wide range of phenotypic variation was detected for all traits (Table 1, Additional file 1: Table S3). The phenotypic BLUEs of all traits along with their variance decreased drastically under stress treatment (Fig. 1). The lowest reductions were observed for Sl and Sdw (7 and 9%, respectively), while the strongest reductions were obtained for Trv and Rdw (50 and 36%, respectively). Considerable reductions in the coefficient of variation (CV) in the osmotic stress treatment were observed for the four image-based root traits, ranging from 4% (Rth) to 51% (Trv) (Additional file 1: Table S3). Furthermore, heritability values were lower under stress (range 0.20–0.68) compared to non-stress conditions (range 0.37–0.78; Additional file 1: Table S4), which is comparable to the manually recorded traits [69]. In both treatments, Trl and Nmr had the lowest heritability. In non-stress conditions, all further traits exhibited heritabilities > 0.5, the highest displayed by Rsr (0.78) and Rth (0.75), while in stress conditions, seven traits showed heritabilities < 0.5. The three traits with higher heritability in stress were Rl, Sdw and Sl (0.68, 0.54 and 0.53, respectively). The lower heritability values and coefficients of variation under imposed stress conditions indicate the complexity of the genotypic response to stress and environmental variation. Further, the reduction response was most pronounced for Trv (50.5%), followed by Rdw and Rl (35.9 and 28.6%, respectively), which suggests that root biomass components were affected most when stress was imposed, while Rth and Nmr were less strong reduced (17.4 and 11.9%, respectively). Least affected were Sl and Sdw (7.1 and 9.1%, respectively), demonstrating most accessions were investing more into shoot biomass under osmotic stress.

**Fig. 2:**
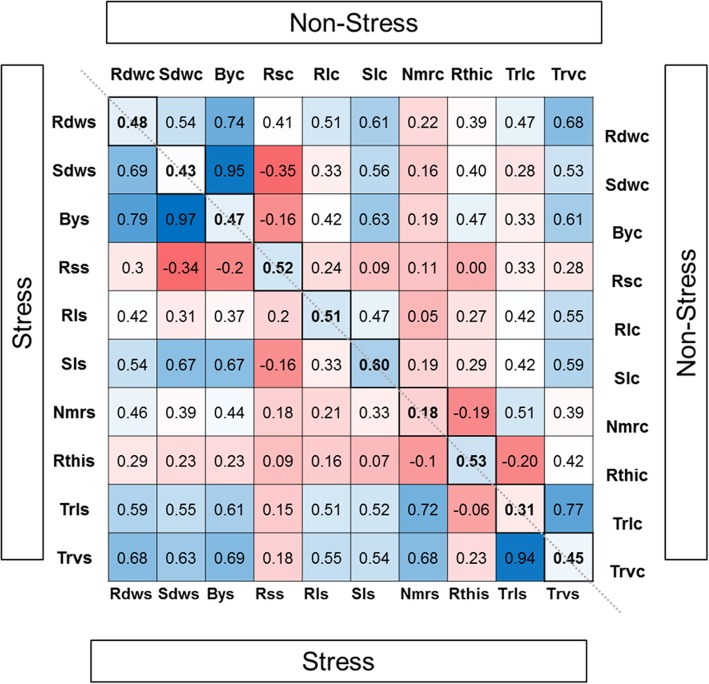
Correlations between root and seedling traits in non-stress or osmotic stress conditions. Correlations are displayed as heatmap and as numerical value. Red = negative correlation, blue = positive correlation. The part above the diagonal presents correlations of traits only within non-stress treatment and below the diagonal only within stress treatment. Along the diagonal correlations between the same trait in both treatment are displayed. Correlations values above 0.2 and below −0.2 are significant (*P* < 0.01)

Significant positive correlations were observed among seedling traits within both treatments, except for Rs (Fig. 2). Among the image-based traits, Trv showed the strongest positive correlation with Rdw across treatments, whereas Nmr and Rth displayed moderate to low-correlation values. In general, stress reduced most of the correlations among various traits but they showed similar relationships under both treatments in line with [21].

### Influence of population structure

The present panel comprised 223 genotypes from a well-studied population and ten additional genotypes. Population structure in this population was extensively investigated by [46] and revealed six subgroups based on row type and geographic origin. In GWAS, the kinship was fully sufficient to control for the confounding effects of population structure. Nevertheless, we have investigated population structure in the extended panel using Structure 2.0. In consistence with [46] the collection clustered according to row type and origin (Additional file 1: Table S5, Additional file 2: supplementary note and Figure S2). All nine additional two-rowed genotypes clustered within group 5 (European two-rowed genotypes), while the only additional six-rowed genotype clustered among the six-rowed European group 9. We tested if the main factor of population structure (row type) affected our phenotypic traits. In control, only one trait was significantly different (Trlc), while under stress, six traits showed differences, in all cases two-rowed genotypes were performing better (Additional file 1: Table S6).

### Genome-wide association scans for root and shoot traits

A set of 4966 mapped and quality-filtered SNP markers were used for GWAS, which were evenly distributed over all seven chromosomes with an average spacing of 4.97 cM. The number of markers varied among chromosomes with a minimum of 483 SNPs on chromosome 1H and a maximum of 967 SNPs on chromosome 5H (Additional file 1: Table S7).

**Fig. 3:**
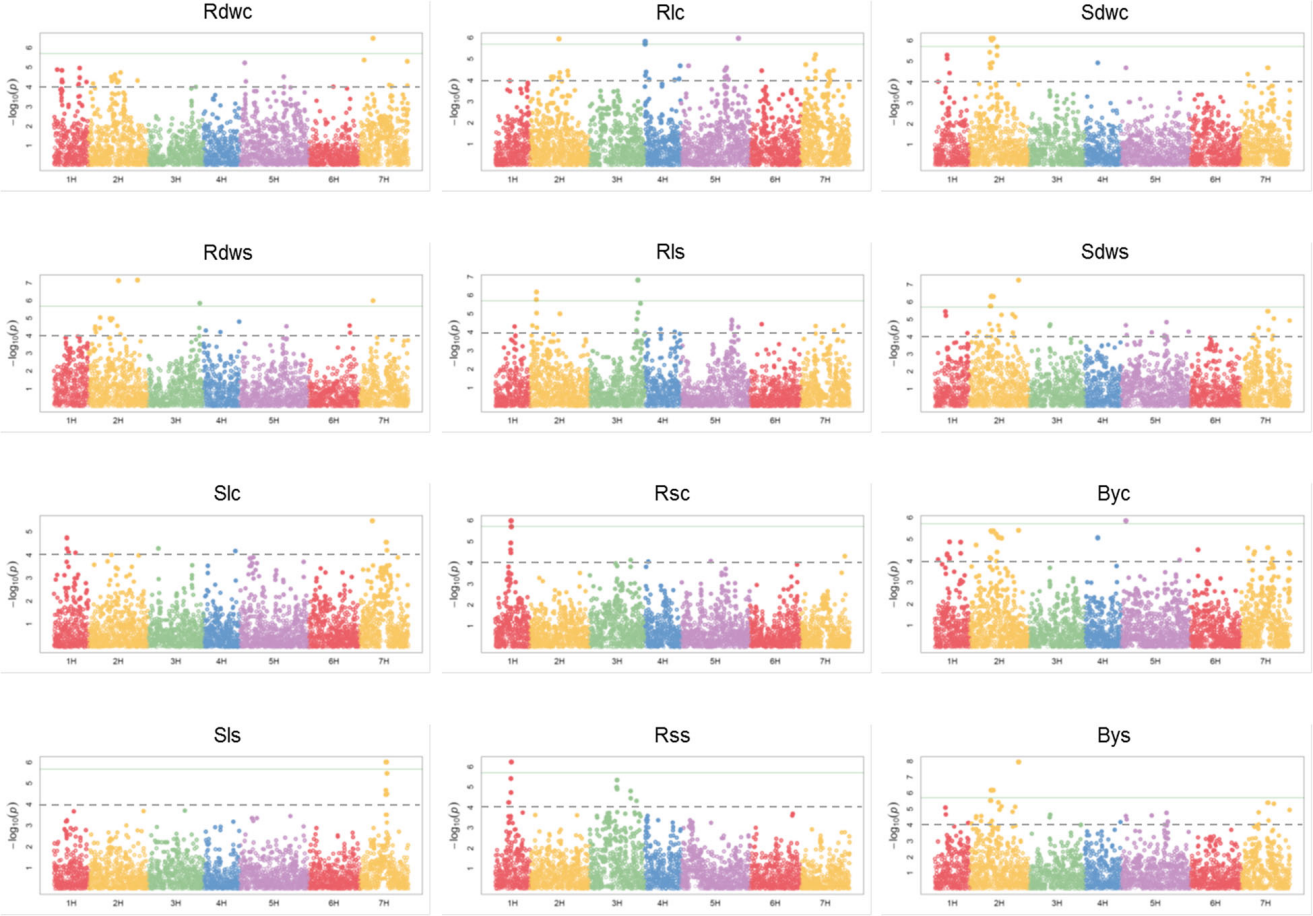
Manhattan plots of 12 out of 21 root and shoot traits. Horizontal axis presents seven chromosomes (1H–7H) of the barley genome. Vertical axis shows -log10(P) values of the marker-trait associations. Horizontal green line shows the threshold value based on FDR (0.05). Additionally, dashed line signifies threshold of -log(p) = 4.0

**Fig. 4:**
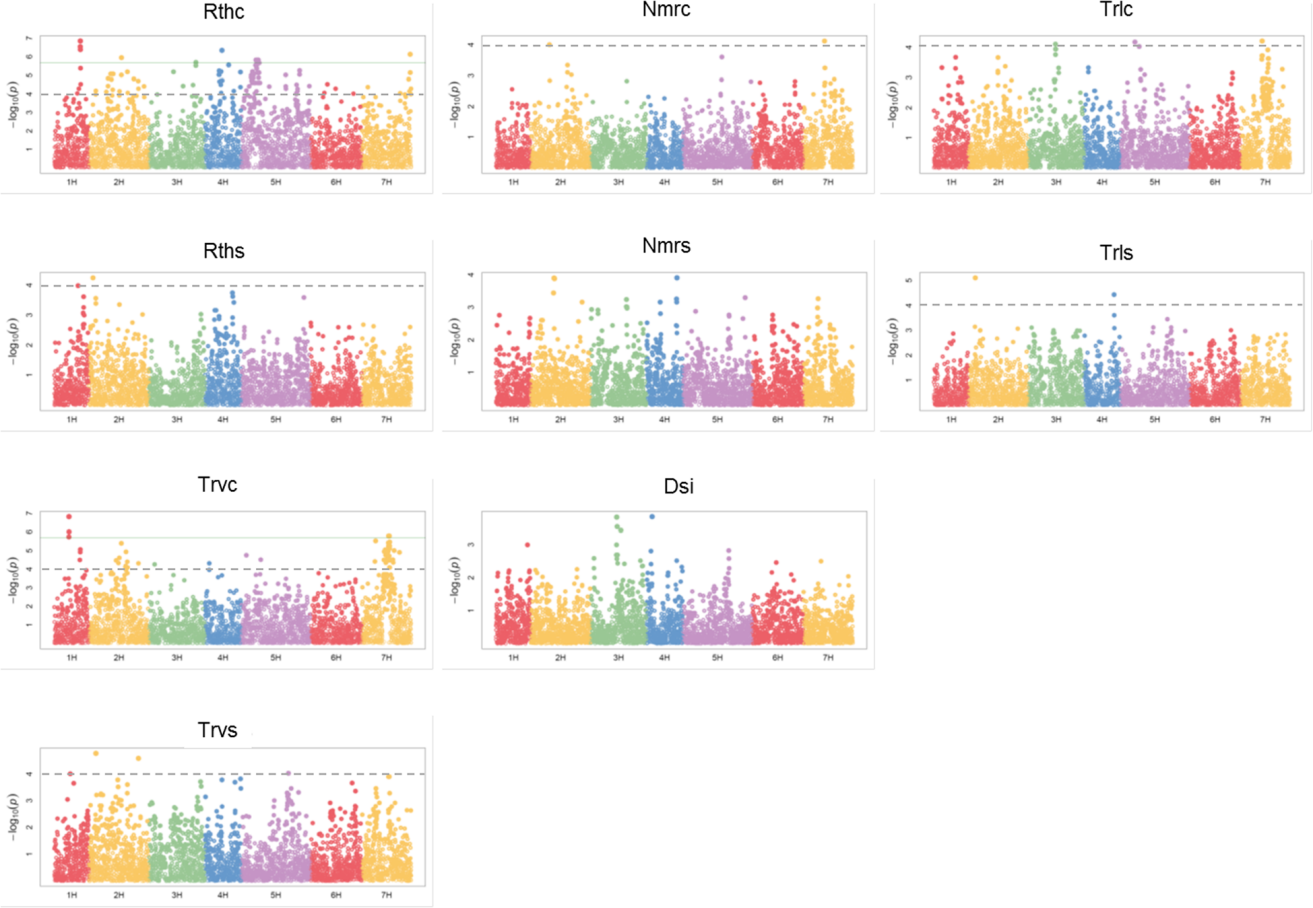
Manhattan plots of 9 out of 21 root and shoot traits. Horizontal axis presents seven chromosomes (1H–7H) of the barley genome. Vertical axis shows -log10(P) values of the marker-trait associations. Horizontal green line shows the threshold value based on FDR (0.05). Additionally, dashed line signifies threshold of -log(p) = 4.0

In total, 519 marker-trait associations were detected (Additional file 1: Table S8), based on 323 SNPs that were associated with one (234 SNPs) and or two and up to five traits (89 SNPs). For all traits analyzed, significant associations were detected for nineteen traits across all seven chromosomes (Figs. 3 and 4), while no significant associations were identified for Nmrs and Dsi.

**Fig. 5:**
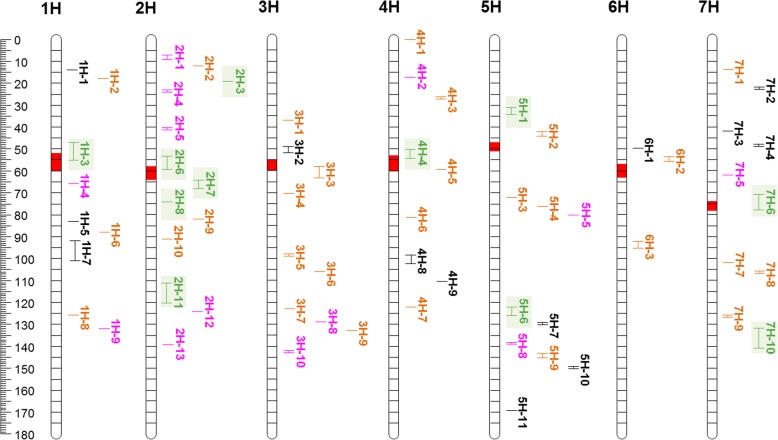
Genetic positions (cM) of 65 QTL regions for root and shoot seedling architecture placed on a schematic map of the seven barley chromosomes along with the corresponding QTL name (see Additional file 1: Table S5 for all details). QTL-hotspots are highlighted in green, root specific QTL in orange, stress-specific QTL in pink and the remaining non-specific QTL in black. Centromeric regions are indicated by red segments

Associated SNPs in close distance were grouped into QTL regions based on the average LD decay of ~ 3.5 cM (data not shown) due to differential LD blocks for individual chromosomes and thus a variable decay across the chromosomes (Additional file 2: Figure S3). This enabled us to detect 65 QTL regions (Fig. 5, Additional file 1: Table S8). The highest number of QTL was identified on chromosomes 2H (13), 5H (11), 3H (10) and 7H (10), and the lowest found on 6H (3). In total, 26 out of all 65 QTL regions correspond to traits from both treatments, while 27 and 12 correspond to traits from non-stress or stress treatment, respectively. This may reflect the reduced heritabilities obtained under stress conditions.

Further, we defined a QTL as a hotspot QTL if at least five out of all ten traits were associated to this region. In total, we identified eleven hotspot QTL and observed a concentration of five hotspots alone on 2H (QTL-2H-3, QTL-2H-6, QTL-2H-7, QTL-2H-8, QTL-2H-11), while the remaining were located on 1H (QTL-1H-3), 4H (QTL-4H-4), 5H (QTL-5H-1, QTL-5H-6) and 7H (QTL-7H-6, QTL-7H-10). All hotspots were associated with traits from both treatments.

### Candidate gene exploration and testing

By educated guess, a list of candidate genes (CGs) was established (Additional file 1: Table S8). We identified developmental, flowering time, stress-related and root-affecting CGs from the recently annotated barley genome assembly [70]. Additionally, root-morphology related gene homologues from Arabidopsis, rice and maize were identified. Thereby, CGs near to our QTL regions were identified which we think are suitable candidates for the associated traits. However, the approach is not accurate, in particular in the centromeric regions where recombination is low and QTL interval is large. To further test polymorphisms within the potential CGs for associations we assembled the polymorphisms of these CGs by using two approaches i) utilization of GBS data for the IPK barley collection [84] and ii) re-sequencing CG fragments within the association panel.

Out of 113 potential CGs (Additional file 1: Table S8), 128 SNPs from 39 CGs were retrieved by GBS approach. However, after filtering for missing data and MAF, 71 SNPs from 29 CGs were tested for associations to our 21 traits with the same model as in GWAS. Association between traits and CG-SNPs with –log(p)-value > 2 were obtained for 17 different CGs (Additional file 1: Table S9), revealing a number of 12 CGs that can be excluded as CGs as they had weaker associations. For nine CGs, the –log(p)-value in GWAS was higher in the corresponding QTL region. This suggests that we miss either the causal SNP or we have not hit the right CG and therefore these nine genes remain potential CGs. Nevertheless, 15 associations corresponding to eight CGs were of similar –log(p)-value as in GWAS (difference maximal 0.3 lower) or of even higher –log(p)-value and therefore support the gene as a CG. The most striking result came from a SNP of *PIN7* associated with Rthc, where the –log(p)-value was 5.92 while in GWAS the highest was only 4.02 in the corresponding QTL region.

In the second approach, fragments of twelve CGs were re-sequenced for the majority of accessions from the GWAS panel (Additional file 1: Table S2). These genes comprised two flowering time genes (*HvPpd-H1* and *HvCEN*) and ten root growth related candidate genes, two of them chosen outside of QTL regions detected in this study. From the twelve CG fragments, eight showed significant trait associations (Additional file 1: Table S10, Additional file 2: Fig. S4), while four genes were rejected as CGs (*HvCEN, ABP1, PRC2, PIN2*). Six CGs had associations similar or higher compared to GWAS and are supported as the right CG (*HvPpD-H1, TRX-m3, HvEXPB1, WOX5, PIN5, HvARF04*). The two other genes remain potential CGs (*HvCKX5, GNOM*).

Two CGs were covered by both approaches, GBS and re-sequencing. Both revealed a rejection of *PIN2* as CG behind root-specific QTL-7H-9 and both suggest a role of *TRX-m3* as a CG for hotspot QTL-2H-6. Nevertheless, some associations from *TRX-m3* based on re-sequencing were similar or higher compared to GWAS and therefore support *TRX-m3* as a CG, while by GBS approach the gene remains a potential CG. However, only one SNP form the gene was available in GBS data compared to seven from re-sequencing.

In summary, 39 CGs were tested for associations to phenotypic traits by one or two of the CG-association approaches. In total, fourteen CGs are supported as candidates, while ten remain potential CGs (association not as high as in GWAS) and fifteen can be rejected as a CG (Table 2).

**Table 2:** Overview of results from candidate gene (CG)-association approach using SNPs from GBS or re-sequencing (RS)

GBS CGs	Annotation in BARLEX	*Alternative names*	Approach	CG status after testing	highest –log(p of CG)	Corresponding QTL region
HORVU1Hr1G057860	cytokinin oxidase/dehydrogenase 1	*AtCKX1*	GBS	potential	2.3	QTL-1H-3
HORVU1Hr1G080950	Glycogen synthase	*HvSSIV*	GBS	potential	2.05	QTL-1H-7
HORVU1Hr1G082250	alcohol dehydrogenase 1	*Adh2*	GBS	supported	2.21	QTL-1H-7
HORVU1Hr1G094980	Early flowering 3	*HvELF3*	GBS	potential	3.26	QTL-1H-9
HORVU2Hr1G013400	pseudo-response regulator 7	*HvPpd-H1*	RS	supported	5.34	QTL-2H-3
HORVU2Hr1G036320	WRKY family transcription factor family protein	*HvSUSIBA2*	GBS	potential	2.03	QTL-2H-6
HORVU2Hr1G080630	14–3-3-like protein GF14-B	*HvGID2*	GBS	potential	3.55	QTL-2H-6
HORVU2Hr1G075240	Thioredoxin superfamily protein	*TRX-m3*	RS	supported	5.65	QTL-2H-6
HORVU2Hr1G075240	Thioredoxin superfamily protein	*TRX-m3*	GBS	potential	2.73	QTL-2H-6
HORVU2Hr1G085910	Zinc finger protein CONSTANS-LIKE 4	*HvCO4*	GBS	supported	4.21	QTL-2H-7
HORVU2Hr1G097490	expansin B4	*HvEXPB1*	RS	supported	2.63	QTL-2H-10
HORVU2Hr1G097380	pectinesterase 11	*PME5*	GBS	supported	3.71	QTL-2H-10
HORVU2Hr1G103780	protein kinase family protein	*HERK2*	GBS	supported	2.73	QTL-2H-11
HORVU3Hr1G075920	Cytokinin dehydrogenase 5	*HvCKX5*	RS	potential	2.27	QTL-3H-4
HORVU3Hr1G085050	WUSCHEL-related homeobox 9	*WOX5*	RS	supported	3.02	QTL-3H-5
HORVU3Hr1G089980	gibberellin 20 oxidase 2	*HvGA20ox3*	GBS	potential	2.51	QTL-3H-6
HORVU3Hr1G094000	Auxin efflux carrier family protein	*PIN5*	RS	supported	3.21	QTL-3H-7
HORVU4Hr1G057550	pseudo-response regulator 7	*HvPRR73*	GBS	supported	6.16	QTL-4H-4
HORVU5Hr1G095530	phytochrome C	*HvPhyC*	GBS	potential	2.15	QTL-5H-2
HORVU6Hr1G058740	cryptochrome 2	*HvCry2*	GBS	supported	2.44	QTL-6H-2
HORVU6Hr1G076110	Auxin efflux carrier family protein|	*PIN7*	GBS	supported	5.92	QTL-6H-3
HORVU7Hr1G074690	glyceraldehyde-3-phosphate dehydrogenase C2	*GAPC3*	GBS	potential	2.16	QTL-7H-6
HORVU7Hr1G120030	Delta(24)-sterol reductase	*HvDIM*	GBS	supported	2.98	QTL-7H-10
HORVU7Hr1G033820	auxin response factor 19	*HvARF04*	RS	supported	3.58	no QTL between QTL-7H-4 and QTL-7H-5
HORVU4Hr1G002880	SEC7-like guanine nucleotide exchange family protein	*GNOM*	RS	potential	2.02	no QTL, between QTL-4H-1 and QTL-4H-2
HORVU1Hr1G039150	CCT motif family protein	*HvCMF10*	GBS	rejected	< 2	QTL-1H-3
HORVU1Hr1G093770	Lysine-specific demethylase 5B	*HvPKDM7–1*	GBS	rejected	< 2	QTL-1H-9
HORVU2Hr1G072750	Protein TERMINAL FLOWER 1	*HvCEN*	RS	rejected	< 2	QTL-2H-6
HORVU2Hr1G113880	AP2-like ethylene-responsive transcription factor	*Zeo1/AP2*	GBS	rejected	< 2	QTL-2H-11
HORVU3Hr1G072810	gibberellin 2-oxidase	*HvGA2ox1*	GBS	rejected	< 2	QTL-3H-3
HORVU3Hr1G090980	gibberellin 20-oxidase 3	*sdw1 / denso*	GBS	rejected	< 2	QTL-3H-7
HORVU3Hr1G106880	Ankyrin repeat family protein / BTB/POZ domain-containing protein	*Uniculme4*	GBS	rejected	< 2	QTL-3H-10
HORVU5Hr1G050510	kinesin 4	*HvBC12/GGD1*	GBS	rejected	< 2	QTL-5H-2
HORVU5Hr1G026780	Auxin-binding protein	*ABP1*	RS	rejected	< 2	QTL-5H-2
HORVU6Hr1G056000	CONSTANS-like 3	*HvCO5*	GBS	rejected	< 2	QTL-6H-2
HORVU6Hr1G057630	Two-component response regulator-like PRR1	*HvPRR1*	GBS	rejected	< 2	QTL-6H-2
HORVU7Hr1G099250	Protein VERNALIZATION INSENSITIVE 3	*PRC2*	RS	rejected	< 2	QTL-7H-7
HORVU7Hr1G110470	Auxin efflux carrier family protein	*PIN2*	GBS	rejected	< 2	QTL-7H-9
HORVU7Hr1G110470	Auxin efflux carrier family protein	*PIN2*	RS	rejected	< 2	QTL-7H-9
HORVU7Hr1G120520	sucrose synthase 6	*HvSuSyII*	GBS	rejected	< 2	QTL-7H-10
HORVU7Hr1G120960	callose synthase 1	*HvGSL5*	GBS	rejected	< 2	QTL-7H-10

If a SNP showed associations with –log(p)-value> 2, the highest association is presented. The column CG status summarizes the result of testing for associations in comparison to GWAS

## Discussion

The present study applied GWAS to root and seedling traits in a diverse spring barley panel of world-wide origin. We demonstrate here that in barley traits related to root and shoot architecture are under the regulation of at least 65 QTL in line with [47]. Chromosome 2H harbors the highest number of QTL. Only a single QTL was specific for shoot architecture (QTL-1H-5), while 31 out of all 65 QTL were linked with both root and shoot traits, indicating linkage and/or pleiotropic effects of these QTL. In total, 34 QTL were exclusively associated with root architecture traits; the majority located on 3H (8), followed by 2H, 4H and 5H (5 QTL each).

By comparing QTL positions found in other studies, we found a number of QTL co-locating with shoot biomass QTL [88] detected in a subset of our collection (QTL-3H-5, QTL-4H-7, QTL-7H-1) and with drought and biomass related QTL in a winter barley collection [89] (Additional file 1: Table S8). Moreover, five root-specific QTL (QTL-2H-4, QTL-2H-10, QTL 3H-4, QTL-5H-6, QTL-5H-9) were co-locating with QTL for agronomic traits in recombinant chromosome substitution lines out of cultivated and wild barley [90], indicating a potential role of these QTL in yield formation.

Further, twelve stress-specific QTL were found on 1H (2), 2H (4), 3H (2), 4H (1), 5H (2) and 7H (1), four of them exclusively found for root traits. To our best knowledge that is the first report on root QTL in barley appearing exclusively under drought stress (QTL-1H-4, QTL-2H-4, QTL-3H-8, QTL-4H-2).

### Flowering time-related genes as candidates for root and shoot architecture at seedling stage under non-stress and osmotic stress conditions

For many QTL a co-localization with flowering time-related genes was observed (Additional file 1: Table S8). This may refer to pleiotropic effects of these genes or to genetic linkage of flowering time genes and the causal gene. A QTL study in *Brassica* revealed a trade of between flowering time and root pulling force (RPF) [91], resulting in co-localization of QTL for flowering time and RPF and also grain yield under both wet and drought conditions in the field. A Wuschel related homeobox (WOX) family protein of the group 13 in Arabidopsis (*AtWOX13*) affected floral transition and was expressed during primary and lateral root formation [92]. It was shown that *HvPpd-H1* directly controls leaf growth in barley [93]. Further, osmotic stress at seedling stage increased the expression of clock-related genes particular for *Ppd-H1, HvPRR73* and *HvPRR95* in barley [94]. Accordingly, ABA-responsive elements (ABRE) were identified in the promoter sequences of clock genes, leading to the assumption that stress regulates their transcription in barley in line with results from Arabidopsis [95]. It was suggested that circadian clock and light regulators are involved in the transcriptional control of stress-response genes. Moreover, Bowman and Scarlett introgression lines varying at *HvELF3* and *HvPpd-H1* possessed a lower biomass under osmotic stress compared with their recurrent parents [94]. These studies demonstrate the role of flowering time genes on seedling growth and stress tolerance. Therefore, flowering time-related genes can be causal genes behind several of our QTL. In total, eleven of them were covered by the one of the two CG-association approaches. Supported as CGs for traits from our study are *HvPpd-H1*, *HvCry2*, *HvCO4* and *HvPRR73,* while *HvGID2*, *HvELF3*, and *HvPhyC* remain potential CGs*.* A role of *HvCEN*, *HvCMF10, HvCO5* and *HvPRR1,* as CGs behind QTL from our study was rejected by the CG-association approaches.

### Genes known to be involved in root growth are located in the vicinity of detected root-specific QTL

Half of all QTL were specific for root architecture and represent the most interesting regions to screen for genes involved in root growth. There is a very limited number of candidate genes presently known to influence root growth in barley.

Half of the twenty identified ARFs in barley [62] are located in the vicinity of QTL identified in our study (Additional file 1:Table S8): hotspots QTL-2H-6 (*HvARF12*), QTL-2H-8 (*HvARF15*), QTL-2H-11 (*HvARF09*) and QTL-5H-1 (*HvARF07*) and root-specific QTL-3H-3 (*HvARF18*), QTL-3H-7 (*HvARF10, HvARF11*), QTL-7H-7 (*HvARF16*) and QTL-7H-8 (*HvARF06*) and the non-specific QTL-1H-7 (*HvARF19*). Unfortunately, none of these ten ARFs was covered by the GBS CG-association approach. However, we re-sequenced *HvARF04* (located outside of detected QTL regions) and detected strong associations to Rthc and Rsc (−log(p) > 3). This undermines the role of ARFs in root architecture and encourages re-sequencing of these genes in our collection.

We further mapped barley homologues to Arabidopsis *PIN-FORMED (PIN)* genes (*PIN2*, *PIN5*, *PIN7* and *PIN8*) [96–100], which encode auxin transporters that control radial root growth [101]. They were mapped in the genomic regions of QTL-7H-9 (Rls and Rsc, third highest allelic effect for Rsc), QTL-3H-7 at 122.6 cM (Rthc), QTL-6H-3 (Rdws and Rthc) and QTL-3H-3 (Rthc, Trlc and Rss, third highest allelic effect for Rss and Trlc), respectively. Mutations in these genes have been reported to cause severe root phenotypes due to de-regulated auxin transport [102]. The QTL-7H-9 region was further reported to be associated with rhizosheath weight in barley [50]. Two of these genes are strongly supported as candidates behind detected QTL in our study by CG-association approaches, namely *PIN5* and *PIN7*, while *PIN2* was rejected as a CG. *GNOM*, a gene involved in the endosomal recycling of the auxin-efflux carrier *PIN1* in Arabidopsis [103] evolved as a potential CG by re-sequencing efforts. Interestingly, for Nmr we detected only two QTL by GWAS. With the GBS CG-association approach, three further QTL for Nmr were detected and the highest association came from *PIN7*.

We found three associations to root traits (Rlc, Trvc, Rthc) for the root-specific QTL-2H-10 containing the CG *HvEXPB1*. This QTL had the highest allelic effect on Trvc and the third highest effect for Rlc. Close by, [50] mapped a QTL for rhizosheath weight in barley at 94.2 cM, highlighting the role of this QTL in different genetic background. However, one SNP of QTL-2H-10 (SCRI_RS_195164) was also associated with biomass under drought stress in winter barley [89]. Accordingly, *HvEXPB1* was associated with Bys and Sdws in the GBS CG-association approach. This indicates an effect of *HvEXPB1* in drought adaptation. Further supported by GBS CG-association approach is *PME5* that was associated exclusively with traits form control conditions (Rls, Slc, Trlc). The gene is encoding for a Pectin Methylesterase that contribute to control of cell wall growth and development [104] and also play a role in the process of root inhibition by aluminum [105]. Recently, [106] showed that *PME5* expression leads to stronger cell walls. Depending on the trait, different genes in the QTL region might be the causal gene. Interestingly, also a QTL for hectoliter weight [90] was mapped to the QTL-2H-10 region and therefore indicates a link of seedling traits to yield formation.

The QTL-3H-5 region was associated with Rsc and Rss (second highest allelic effect). A QTL for Rdw (96.7 cM) in close vicinity was identified earlier by [59] using 223 genotypes that are part of our GWAS panel. A homologue to the Arabidopsis auxin transporter gene *AUX1,* which is involved in lateral root development [107] and a homologue to *WUSCHEL-RELATED HOMEOBOX5 (WOX5)* - a gene related to root organogenesis - are located in very close proximity to QTL-3H-5. Also, the flowering time gene *HvCMF1* is located within the QTL region and may cause pleiotropic effects on root growth. Only *WOX5* could be tested in the CG-association approach and is supported as CG by re-sequencing.

In the recent past, several candidate genes affecting root development were isolated from cereals. The predicted position of *root hairless 3* (*RTH3*) [108] is in the region of hotspot QTL-4H-4, where five root and shoot traits showed significant associations. Within the vicinity of root specific QTL-4H-7, a CG is located with high sequence similarity to *adventitious rootless1 (ARL1)* and *seminal roots, crown rootless1* (*CRL1*) in rice [109, 110] and *rootless concerning crown* (*RTCS*) in maize [111]. These genes affect the initiation of adventitious and crown root formation, respectively, by regulating polar auxin transport. The region of QTL-4H-7 might also affect above ground trait architecture indicated by the co-localisation with a shoot biomass QTL [89].

The QTL-5H-2 was detected for Rthc, Rlc, Trlc and Trvc and showed the highest allelic effect for Trvc. Interestingly, in this QTL region [51] located QTL for RDW, Rl and Rs. Further, seven of all 44 SNPs from that QTLregion were also associated with biomass in control or in drought conditions in winter barley [89]. QTL-5H-2 coincides with a QTL for tiller number found in the smaller subset of the collection [71] but none of the SNPs from QTL-5H-2 is identical to the 26 significant SNPs from [88]. Therefore, different CGs might be causal for these traits but in the centromeric region resolution is low and the right candidate hard to identify. The region contains many genes including flowering time genes such as *HvCMF13, HvCO3, HvPRR95* and *HvPhyC*. Using the GBS CG-association approach, *HvPhyC* evolved as a potential CG, while the other three CGs were not tested for associations.

Another root-specific QTL-6H-2 harbors three flowering time genes. From *HvCry2, HvCO5*, and *HvPRR1*, *HvCry2* is supported by GBS CG-association approach as a candidate, while *HvCO5* and *HvPRR1* were rejected.

The root specific QTL-7H-1 associated with Rlc, RDWc and Rthc harbors the *waxy* locus encoding for a *granule-bound starch synthase I* (*GBSS I*), which catalyzes the synthesis of amylose synthesis in developing grains [112]. As a secondary effect, developing seedlings might have better conditions for early growth as they feed from the starch stored in the grain. Accordingly, a QTL for early shoot biomass was detected in the smaller two-rowed subset of the present collection [88]. However, *HvWAXY* could not be tested for associations and should be re-sequenced to unravel its role as a CG.

Hotspot QTL for root and shoot seedling architecture under contrasting growth conditions

The present study showed the occurrence of eleven QTL hotspots for root and shoot traits from both treatments on five chromosomes. QTL hotspots for yield-related traits were reported in barley [e.g. [113–116] ], while for root and shoot traits at seedling stage, information is limited. In chickpea, [117] reported one QTL hotspot for root traits including root length density, suggesting that pleiotropic effects for root architecture traits may exist as a common feature in crop plants.

In our study, hotspot QTL-1H-3 was associated with six traits. For Rsc, Rss and Trvc, this QTL had the highest allelic effects for Rsc and Rss. Seven SNPs of QTL-1H-3 were associated with seedling biomass in control conditions or with DSI in winter barley [89], indicating this genomic region has an impact on plant growth in broader genetic background, at different growth stages and under contrasting growth conditions. Located within QTL-1H-3 region resides GA INSENSITIVE DWARF 1 (*HvGID1*) affecting plant height and growth, and therefore representing a strong candidate for this hotspot. Also, the flowering time gene *HvCMF10* is located in the region and may have pleiotropic effects. Further, the *SIX-ROWED SPIKE3* (*Vrs3*) is located in this region. This gene has been reported to cause effects on lateral grain size and grain uniformity [118, 119] and acts as a transcriptional activator of row-type genes like *Vrs1* and *int-C* which affects tillering and shoot branching [120]. However, a role of *Vrs3* in modifying root traits is unknown. A root-related CG represents *AtCKX1*, a cytokinin oxidase/dehydrogenase. Cytokinins have an important but opposite role in growth of roots and shoots [121]. They modulate root elongation and the number of lateral roots [122]. By the GBS CG-association approach, *AtCKX1* remains a potential CG, while *HvCMF10* was rejected. *HvGID1* and *Vrs3* were not tested.

Hotspot QTL-2H-3 harbors the major locus of flowering time in spring barley *HvPpd-H1*, a PSEUDO-RESPONSE REGULATOR [62]. In previous studies, the association of *HvPpd-H1* with many agronomic traits has been well elaborated [72, 123–127]. Recently, *HvPpd-H1* was shown to directly influence leaf growth by it’s involvement in leaf meristem activity under long-day conditions [93, 128]. Our GWAS panel differs for photoperiod sensitivity [72] and our experiments were performed under long day conditions [69]. The re-sequencing CG-association approach of a 1367 bp gene fragment supported *HvPpd-H1* as the causal gene behind QTL-2H-3. High associations, including the functional SNP [129], were found for root and shoot biomass under osmotic stress and non-stress conditions, demonstrating the effect of *HvPpd-H1* also on root growth which was unknown so far. Nevertheless, not all traits from this hotspot, namely Rthc, Trls and Trvs, were associated with SNPs within *HvPpd-H1*, indicating that additional unknown genes in this region might cause the effects for these three traits.

Among the five QTL hotspots on 2H, QTL-2H-6 and QTL-2H-7 are located in the peri-centromeric region which has reduced recombination [130] and harbors several CGs known to affect root morphology. QTL-2H-6 was the QTL with the highest allelic effects for Byc, Bys, Nmrc, Rthc, Sdwc and Sdws and the second highest effects for Rdws and Rlc. In accordance, [51] reported a major QTL for shoot dry weight “QSdw.2H.a” and QTL for RDW and RS in this region. Additionally, a QTL for osmotic adjustment was mapped by [89] in winter barley to the same region. In the region of QTL-2H-6, resides a barley homologue to the *Arabidopsis thaliana* transcription factor *SHORT ROOT* (*SHR*) [131], which regulates radial patterning of the ground tissue in roots and modifies root morphology. Two further genes are located in this region, which are homologous to Prolyl-4-hydroxylase (*AtP4H*) and Thioredoxin-m3 (*Trx-m3*) in Arabidopsis [132, 133] linked in root meristem and root hairs development.. Furthermore, the gene *HvHOX2* is located in this region, encoding for homeodomain-leucine zipper (HD-Zip) I transcription factor regulating in cereals the plant’s response to abiotic stresses, including osmotic stress [134]. In addition, auxin transcription factor *HvARF12* is located within QTL-2H-6 region and the major flowering time gene *HvCEN*. The presence of many plausible CGs in the centromeric region harboring QTL-2H-6 indicates different genes might be causal for the different traits in that hotspot. By the re-sequencing CG-association approach, we rejected *HvCEN*. An unexpected, striking result came from *Trx-m3* that is supported by the re-sequencing CG-association approach as potential causative gene for Rthc and Byc (the QTL had the highest allelic effects for these traits) and for Trvc. None of the other CGs was tested for associations.

The hotspot QTL-2H-7 is associated with six root and shoot traits. Very close to this region, a QTL for vegetative biomass at 68.6 cM was identified by [89]. The region harbors *HvCO4*, a flowering time gene. Another interesting CG represents *MutS HOMOLOG1* (*MSH1*). The MSH1 protein targets plastids and mitochondria and is involved in genome stability. However, surpression or loss of the gene has huge impact on altered development and enhanced growth vigour by epigentic changes [135, 136]. There is implication that MSH1 acts as environmental sensor and stress signal transmission [137]. Nothing is known yet about phenotypic effects of natural variation of *MSH1.* A QTL hotpsot for early seedling growth under control and osmotic stress conditions in its vicinity encourages future research in this direction. *MSH1* was not tested in our CG-association approaches. However, *HvCO4* evolved as a strong candidate for Rthc by GBS CG-association approach.

Another hotspot QTL associated with six root and shoot traits is QTL-2H-8. In close proximity a biomass QTL was found in the study of [89]. A candidate might be *HvARF15*, an auxin transcription factor that could not be tested as a CG.

Five traits associated to the hotspot QTL-2H-11 with highest significance for Sdwc and the second highest allelic effects for Sdws and Trvs. Accordingly, QTL for biomass and DSI were mapped to this region by [89]. Furthermore, a SNP of QTL-2H-11 associated with Rthc (SCRI_RS_200949), was detected as a QTL for rhizosheath weight [50], indicating a high relevance of this hotspot for root traits. One CG for this locus represents *HvAPETELA2* (*HvAP2/EREBP*) that affects pollination by expanding the lodicule size in the floret; in mutants the density of the grains in the spike increased along with a decrease of the internode length of the stem and spike [138, 139]. It is possible that the same gene also affects below-ground root traits by modifying traits like root weight or thickness. AP2/ERF transcription factors are further known to be induced by osmotic stress [140]. Another CG for this locus represents *HvARF09,* an auxin transcription factor. Moreover, a gene with role in cell elongation *HERK2* is located in this region, having an antagonistic role in root and hypocotyl elongation [141]. This gene was supported by the GBS CG-association approach.

The only hotspot QTL of 4H (QTL-4H-4), is among the most three important QTL for Byc and Sdwc in terms of allelic effects and is harboring the flowering time genes *HvPhyB* and *HvPRR73.* Further, the region contains the root gene *RTH3*. QTL for root shoot ratio [51] and for for osmotic adjustment [89] were reported for this region. Therefore, the region may harbor also stress tolerance related genes. However, the centromeric region contains many genes hampering the identification of the underlying gene(s) of this locus. By the GBS CG-association approach *HvPRR73* is supported as a CG for Rth.

Hotspot QTL-5H-1 is among the three most important QTL for Byc, Rdwc and Sdwc in size of allelic effects and further coincides with a tiller number QTL in the vegetative stage in a subset of our collection [88] and with a QTL for biomass and DSI in winter barley [89]. One CG represents *HvNCED1* that is located in very close proximity to QTL-5H-1. The nine-cis-epoxycarotenoid dioxygenase (*NCED*) catalizes the rate-limiting step in ABA biosynthesis [142]. Aside from auxin, ABA influences root growth by cell divison and elongation [143]. Further, ABA is involved in controlling root and shoot growth under drought conditions [144]. Another CG for this hotspot is *HvARF07,* an auxin transcription factor. None of the two CGs were tested.

In our study hotspot QTL-5H-6 had the the third highest allelic effect on Sdws. *VRN-H1*, a major gene determining the requirement of vernalization, is located within the region. Further, *VRN-H1* coincided with the chromosomal location of a yield and biomass QTL in barley under drought conditions in Syria [145], while [68] reported that *VRN-H1* exhibits pleiotropic effects on root and plant morphology in wheat and barley. However, also the root-specific *HvWAK1* is located in this region. This gene represents a further interesting candidate for re-sequencing.

Hotspot QTL-7H-6 was associated with eight traits from both treatments. This region contains *HvCO1,* the barley homologue to *CONSTANS* in Arabidopsis [146, 147]. Expression of *HvCO1* accelerates inflorescence development and stem elongation [148]. It is tempting to speculate that this gene might also affect root architecture. The barley clock homologue *HvCCA1/HvLHY* [149] is also located within the QTL-7H-6 interval. This genomic region was further associated with multiple traits identified in previous studies in barley, like tiller number, phase duration, rhizosheath weight and biomass [50, 72, 89, 123]. Tillering and plant height is affected by *MONOCULM 1* (*MOC1*) which is located within this region [150]. Another CG could be an orthologue of *OsGAPC3*, encoding for glyceraldehyde-3-phosphate dehydrogenase shown to enhance salt tolerance in rice seedlings [151]. Therefore, co-localization of many agronomic QTL in that hotspot region might very well arise from different CGs depending on the phenotypic trait. By GBS CG-association approach the supposed barley orthologue of *OsGAPC3* remains a potential CG.

Hotspot QTL-7H-10 showed the third highest allelic effect for Rthc. Two SNPs of the region at 140.8–140.9 cM were associated with Byc, Bys, Rthc and Sdws, and at the same position (but a different SNP) a strong QTL for vegetative biomass was identified in a subset of the collection [88] and in other collections [50, 89]. QTL for tiller number [123] in the same region indicate the importance of the region for biomass related traits across different germplasm pools and growth stages. The region harbors several growth-related CGs such as *HvDIM, HvGsl5, HvSuSyII* and an *ent*-copalyl diphosphate synthase. Interestingly, *HvDIM* is supported as a CG by the GBS CG-association approach, while *HvGsl5* and *HvSuSyII* were rejected. However, SNPs from *HvDIM* detected in the GBS CG association approach were associated with Sls, SLs and Trlc, although no significant QTL was detected by GWAS for these traits in the region of QTL-7H-10.

Stress-specific QTL regions at seedling stage

In total, twelve QTL were classified as stress-specific in our study. Six coincided with QTL found by [89] using a vegetative drought at the tillering stage (Additional file 1: Table S8).

The first was QTL-2H-1, significant for Bys, Rls and Rths and possessing the highest allelic effect for Rths. QTL for biomass in drought and well-watered conditions were found in close proximity [89]. A potential CG represents the *pglcat6* gene, encoding for a beta-3-glucuronyltransferase. In Arabidopsis, a beta-3-glucuronyltransferase was shown to be involved in cell elongation at the seedling stage [152].

QTL-2H-4 was significant for Rdws and Rls and co-locates with a QTL for the DSI [89]. QTL for harvest index and TKW under rain-fed conditions were mapped to the same position [90], revealing a suggestive role of this QTL under natural dry conditions. A heat stress transcription factor (HSF) *HsfA2b* is located in very close proximity to this stress-specific QTL. HSFs have a regulating role for stress-responsive genes under various types of abiotic stresses such as heat, drought or salinity [153].

QTL-2H-5 was associated with Sdws, Rdws, Bys and co-locates with a QTL for DSI in winter barley [89]. It coincides with a QTL for biomass under drought in a subset of our mapping panel (Dhanagond et al.; in preparation). The region is promising to harbor CGs for drought tolerance. Among the annotated genes in this QTL region, we identified *HvHAK2* as a CG, a potassium transporter of the KUP6 family, which are key factors in osmotic adjustment [154].

QTL-2H-12 was exclusively linked with Sdws in our study but co-locates with a QTL for vegetative tiller number in well-watered conditions in a smaller subset of the present collection [88] and with a QTL for biomass in well-watered conditions in winter barley [89] and therefore might not be truly stress-specific.

QTL-5H-5 was associated with Bys and Sdws and was for both traits among the most three important QTL in terms of allelic effects. Moreover, QTL for biomass under drought stress and for osmotic adjustment were identified in this region [89], thereby raising interest in the underlying genes. However, the corresponding SNP is not validated in the physical map of barley and therefore identification of CGs is hampered.

QTL-5H-8, trait specific for Rls, co-locates with a biomass QTL during drought stress in a subset of the collection (Dhanagond et al.; in preparation). In winter barley, QTL for DSI and biomass and for osmotic adjustment were mapped to the same region [89], undermining the importance of this genomic region for stress tolerance. In the vicinity of this QTL is located *Dhn9,* a gene known to be involved in drought tolerance. Dehydrins are a group of late embryogenesis abundant proteins forming in response to drought [155].

A QTL for Rs was identified by [51] within the vicinity of QTL-7H-5. Very close to the QTL maps the *Vegetative to Reproductive Transition gene 2* (*HvVRT-2*), a SVP-like gene delaying floral transition and induced by cold [156]. However, a role in drought response is unknown. In proximal position to QTL-7H-5 resides *HvABF2* belonging to a subclass of bzip- transcription factors that regulate ABA stress response [157]. Expression of ABFs is induced by abiotic stress [158] and it was shown that overexpression of ABF2 leads to slower germination and growth of young seedlings and further to increased drought, heat and salt tolerance in Arabidopsis [159]. This gene is therefore a strong CG for this locus but was not tested in the CG-association approaches.

The remaining five stress-specific QTL did not co-locate with QTL from other studies and might be specific for seedling stage stress tolerance or specific for our spring barley panel.

## Conclusions

This study demonstrates that in barley a wide range of natural genetic variation exists for root and seedling traits that can be exploited for crop improvement. In total, 65 genomic regions underlying root and shoot architecture and osmotic stress tolerance at seedling stage were identified by GWAS. This demonstrates the rich genetic diversity of the well-characterized spring barley mapping panel. However, future studies should address winter barley to compare the genetic architecture across both germplasm pools. In total, 14 CG were supported by CG-association approaches. Findings suggest that the flowering time genes *HvPpd-H1, HvCry2, HvCO4* and *HvPRR73* are potentially involved in root and shoot formation and osmotic stress tolerance. A direct role of *HvPpd-H1* on root growth should be investigated. The co-localization of detected root QTL to auxin response factors and PIN-FORMED genes and the support for three of these genes (*HvARF04, PIN5, PIN7*) from CG-association approaches encourages further research on their role in barley. Moreover, further root-related genes were supported: *HERK2, HvEXPB1, PME5,* and *WOX5*. *Trx-m3* was supported as CG for Rthc and Byc within the most important QTL region for these two traits. Our results highlight these candidates as genes with future potential in breeding for enhanced early vigor and a better root system. QTL regions were identified for osmotic stress tolerance and potential candidates comprise *HsfA2b*, *HvABF2, HvHAK2* and *Dhn9*.

## Additional files

Additional file 1: **Table S1.**Overview of 233 genotypes used in this study, their accession numbers, collection name, origin, row type and biological status. **Table S2.**Candidate genes re-sequenced in the association panel with primer combinations used. Details are provided for amplified fragment sizes, primer combinations and annealing temperature **Table S3.**Summary statistics of ten root and shoot seedling traits and for DSI. **Table S4.**Estimates of variance components and broad sense heritabilities under non- stress and 15% PEG drought stress conditions. **Table S5.**Analysis of Molecular Variance (AMOVA) for the 233 diverse barley genotypes based on K = 9, employing 6019 SNP markers. **Table S6.**Influence of row-type on the phenotypic traits: t-test results and phenotypic mean values for both row-types. **Table S7.**Distribution of mapped SNPs across seven barley chromosomes, coverage and polymorphism information content (PIC). **Table S8.** Summary of the significant QTL. Trait names, SNP marker names, chromosome, genetic positions, *p*-value of the associated SNP, minor allele frequency (MAF), R^2^-value of the SNP model with and without model, allelic effects, −log(p)-value, QTL-names, and candidate genes along with the marker physical map positions are provided. **Table S9.** Summary of significant SNPs from candidate genes covered by GBS. **Table S10.**Summary of significant SNPs from candidate genes covered by re-sequencing. (XLSX 181 kb)

Additional file 2: **Figure S1.** Examples of scanned root images from individual plants. **Figure S2.** Concatenated split network tree for the collection of 233 accessions based on 6019 SNP markers. **Figure S3.** LD pattern along the individual chromosomes of barley. **Figure S4.** Schematic representation of the eight re-sequenced candidate genes models. (DOCX 3427 kb)

## Abbreviations

BLUEs: best linear unbiased estimates
CG: candidate gene
FDR: false discovery rate
GWAS: genome-wide association study
LD: linkage disequilibrium
QTL: quantitative trait locus
SNP: single-nucleotide polymorphism
