# Supplementary material for: Genome-wide association mapping in a diverse spring barley collection reveals the presence of QTL hotspots and candidate genes for root and shoot architecture traits at seedling stage
Source: BMC Plant Biol. 2019 May 23;19:216. doi: 10.1186/s12870-019-1828-5 (PMC6533710; doi:10.1186/s12870-019-1828-5)
Supplement: Supplementary file 2 — Figure S1. Examples of scanned root images from individual plants. Figure S2. Concatenated split network tree for the collection of 233 accessions based on 6019 SNP markers. Figure S3. LD pattern along the individual chromosomes of barley. Figure S4. Schematic representation of the eight re-sequenced candidate genes models. (DOCX 3427 kb) [file 12870_2019_1828_MOESM2_ESM.docx]

**Genome-wide association mapping in a diverse spring barley collection reveals the presence of QTL hotspots and candidate genes for root and shoot architecture traits at seedling stage**

Adel H. Abdel-Ghani, Rajiv Sharma, Celestine Wabila, Sidram Dhanagond, Saed J. Owais, Mahmud A Duwayri, [Saddam A. Al-Dalain](http://www.pubfacts.com/author/Saddam+Aref+Al-Dalain), Christian Klukas, Dijun Chen, Thomas Lübberstedt, Nicolaus von Wirén, Andreas Graner, Benjamin Kilian, Kerstin Neumann

Supplementary Data

Supplementary note on population structure in the GWAS panel

Fig. S1

Fig. S2

Fig. S3

Fig. S4

**Population structure and Analysis of Molecular Variance (AMOVA)**

To estimate the number of groups in our association panel, population structure analysis (Structure 2.0; Pritchard et al) was run assuming *K*=1 to *K*=20. Analysis of population structure yielded nine sub-populations (*K*=9) as LnP(D) values reached a plateau at *K* = 9 which was also supported independently by Split Tree. The spring barley collection is clearly separated into two distinct groups according to the row type (2-rowed and 6-rowed) with very few exceptions. The two main clades were further divided into nine clusters (Figure S2). High population variation was found within barley sub-populations, but considerable variation among sub-populations still exists. The variance components of within and between populations detected with AMOVA were 71.6% and 28.6% of the total variance, respectively, which were both significant at *P* ≤ 0.001 (Additional file 1, Table S5). Excluding the admixed group (subpopulation 8), a little bit lower within population variation were observed (60.1%), and among populations' variations were increased to reach 39.9%. Grouping of sub-populations is clearly dependent on row-type and origin of the genotypes. The two-rowed barleys included two big subpopulation originated from Europe (sub-population 2 and sub-population 5), and one small sub-population (sub-population 6 with 4 genotypes) originated from WANA. Six rowed barley was sub-structured into two major groups: subpopulation 4 (mostly originated from EA and admixed with 5 genotypes from WANA) and sub-population 9 (mostly originated from EU), and another three small groups: sub-population 1, 3 and 7 with 12, 3 and 7 genotypes respectively originated from WANA and AM. Further, sub-population 8 was a admixture of two and six rowed barley with no clear pattern of clustering by origin for this group.

**Phenotypic differences within the groups**

We tested the sub-populations for phenotypic differences and observed that six-rowed barley showed a tendency to more vigorous root system as compared to two-rowed barleys subpopulations (Additional file 1, Table S6). Six-rowed genotypes of sub-population 1 consistently had more vigorous roots system than genotypes belonging to other sub-populations under both optimum and PEG-induced drought conditions. Another interesting two-rowed subpopulation was found in sub-population 6, having a medium vigorous root system under optimum and PEG induced drought stress conditions, represented by the traits RDW, TRL and TRV. Contrastingly, three European sub-populations (2, 5 and 9) and a sub-population (7) with AM and WANA origins showed a relatively poor root performance compared to all other sub-populations under optimum and PEG-induced drought stress conditions. Other sub-populations (3 and 4) exhibited medium vigorous root systems.


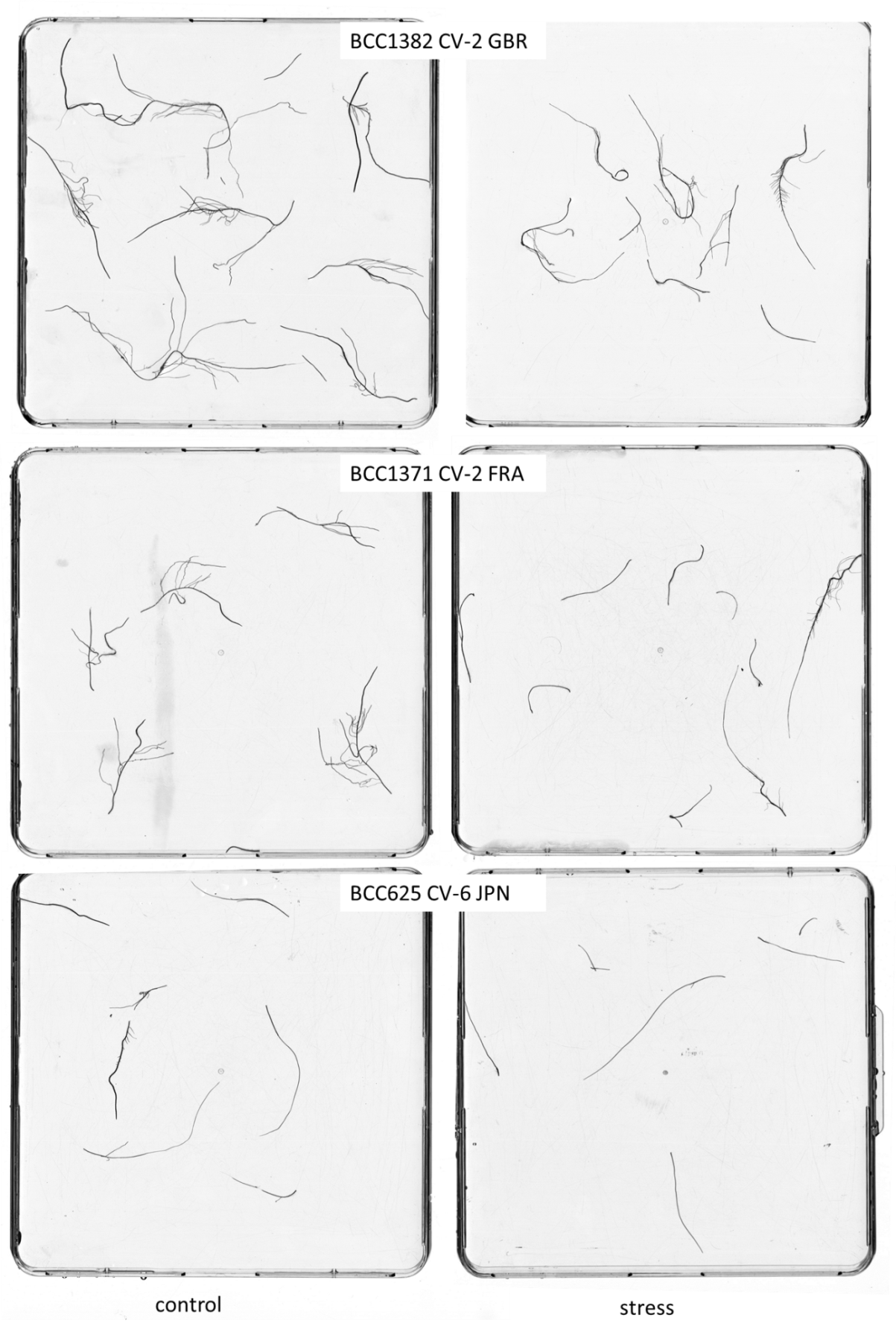


**Fig. S1:** Image scans of the roots. Examples of scanned roots from individual plants of three genotypes with small, medium and large root systems: on the left side are shown roots from osmotic stress treatment and on the right, rots from the non-stress treatment.


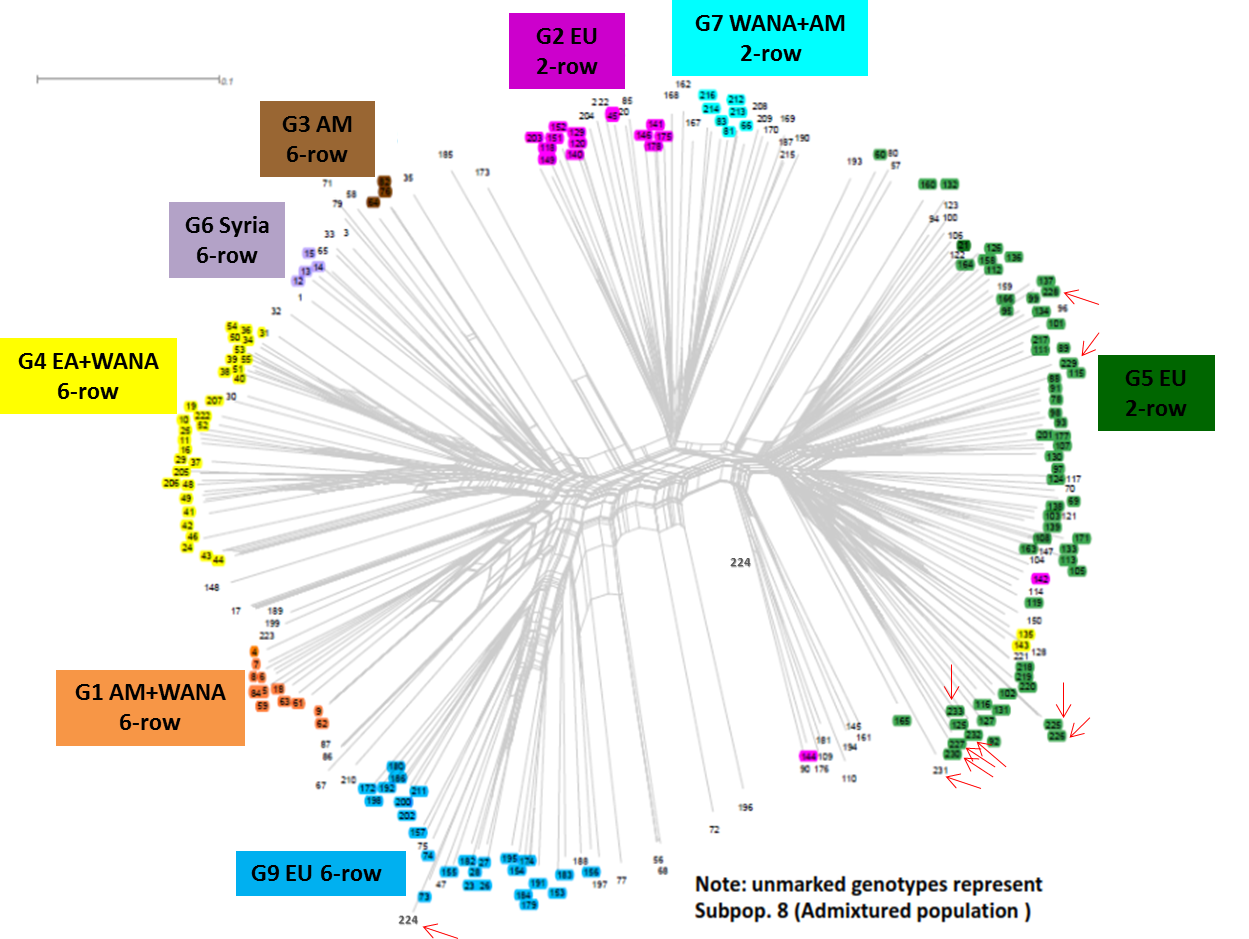


**Fig S2:** Neighbor Net planar network tree for the collection of 233 accessions based on 6,019 SNP markers with Hamming distances (uncorrected-P). The colors indicate the 9 different sub-populations identified by STRUCTURE 2.0. The red arrows indicate the ten additional genotypes compared to the widely used panel of (Pasam et al. 2012).


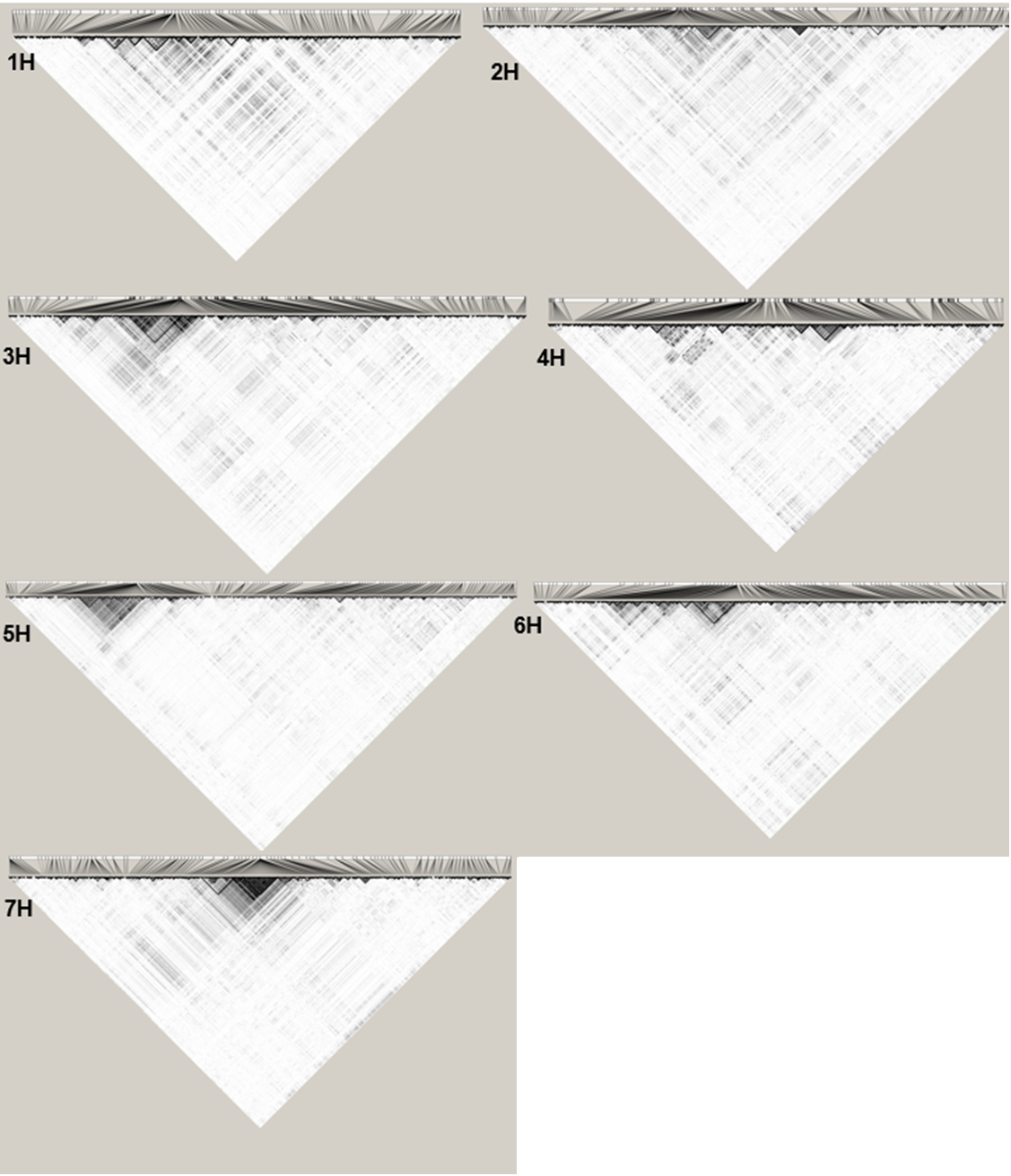


**Fig S3:** LD pattern along the individual chromosomes of barley. High LD regions are shown with grey and black colors. Solid spine of LD method was used to define the LD blocks as implemented in the Haploview version 4.2 (Barret et al. 2005).

**Fig S4** Schematic representation of eight re-sequenced candidate genes models, where in the CG-association approach significant associations were found. All identified SNPs within the re-sequenced region are indicated and those with -log(p)-value >2 in CG-association approach are highlighted in yellow (see Table S10) for more details.
